# Surgical Treatment of Lip Pits in Van der Woude Syndrome: A Preliminary Retrospective Study of 24 Patients

**DOI:** 10.34763/jmotherandchild.20242801.d-24-00020

**Published:** 2024-06-19

**Authors:** Łukasz Wieprzowski, Zbigniew Surowiec, Ewa Sawicka, Andrzej Brudnicki

**Affiliations:** Department of Maxillo-facial Surgery, Clinic of Pediatric Surgery, Institute of Mother and Child, Kasprzaka Street 17a, 01-211 Warsaw, Poland

**Keywords:** Van der Woude syndrome, cleft lip and/or palate, primary cleft repair, lip pits

## Abstract

**Background:**

Van der Woude syndrome (VWS) is a rare congenital malformation characterized by lower lip pits among patients with a lip and/or palate cleft. It is transmitted by an autosomal dominant inheritance with variable expressivity.

**Methods:**

The study group consisted of 24 consecutive patients (13 males and 11 females) with VWS operated on at a single center between 2009 and 2022. They suffered from: bilateral cleft lip and palate – 6 patients; unilateral cleft lip and palate – 9 patients; cleft lip – 1 patient; and isolated cleft palate – 8 patients.

**Results:**

In 16 (66%) cases pits of lower lip occurred on both side of midline, while in 8 (34%) the pits were detected unilaterally. The primary cleft repairs were performed according to one-stage principle at the mean age of 8.6 months (SD 1.4, range 6–12). In all patients lower lip pits repairs were performed after the primary cleft repairs as a separate procedure at the mean age of 37 months (SD 11.3 range 14–85). The mean number of all primary repairs of the syndrome—both cleft defect and lower lip pits repairs—was 2.46. Nine patients (37.5%) required additional secondary corrections of the lower lip due to the poor aesthetic post-operative outcome.

**Conclusions:**

The frequent need for secondary corrections of residual lower lip deformities indicates the considerable difficulties in obtaining a satisfactory outcome of the repairs to lip pits caused by VWS. The average number of the primary surgical interventions in evaluated material remained low.

## Introduction

**Figure 1.**
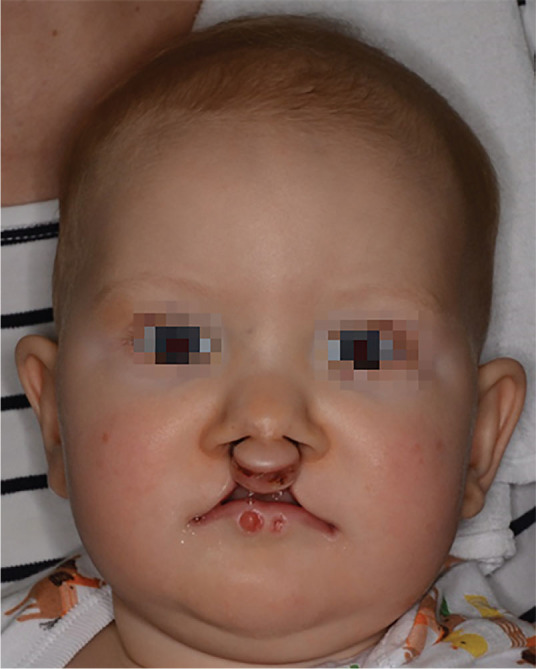
An 8-month-old patient suffering from VWS, before primary BCLP repair.

**Figure 2.**
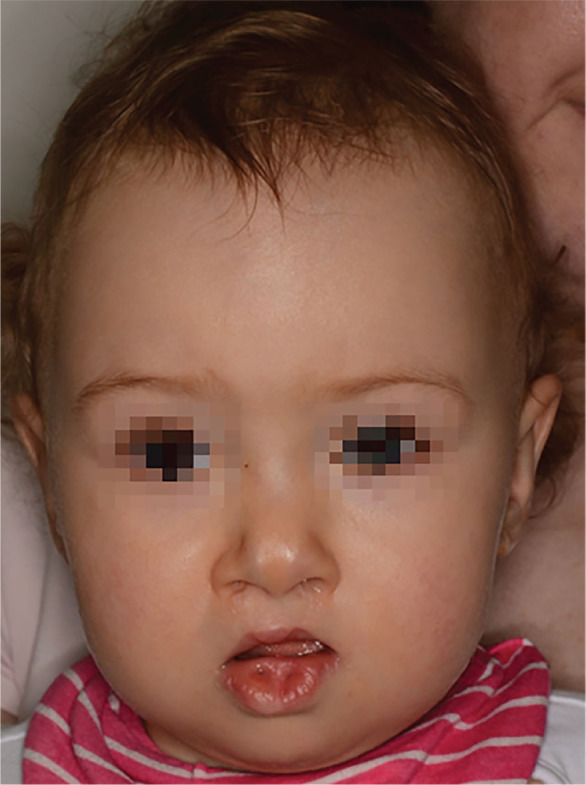
A one-year-old patient suffering from VWS, after one-stage primary repair of BCLP and before lower lip pits repair.

Van der Woude syndrome (VWS) is described as lower lip pits, sinuses or conical elevation in children with cleft lip and palate or isolated cleft palate (Figs. 1, 2). The first to describe congenital lip sinuses was Jean Nicolas Demarquay in 1845 [1], but the syndrome is named for Anne Van der Woude, who in 1954 was the first to combine congenital pits of the lower lip with cleft lip and palate. This autosomal dominant malformation accounts for approximately 2% of all cleft cases [2], and although it is claimed to be the most frequent cleft syndrome [3] it is in fact very rare. The incidence of VWS varies from 1:100 000 to 1:40 000 still born or life births [2,4].

This genetic defect of the lip pits is caused by a micro deletion on chromosome bands Iq32-q4 [5,6]. The cause of VWS has recently been identified as mutation in interferon regulatory factor 6 (IRF 6), which may lead to hypodontia and other dental anomalies [7,8,9]. Malformations associated with VWS include: syndactyly of the hands, clubfeet, genitourinary abnormalities, and cardiovascular anomalies [3].

The most common signs of VWS are bilateral sinuses of the lower lip, placed near the midline in a symmetric or asymmetric fashion. The lower lip pits can also be unilateral. The lip pits vary in location on the lower lip (from the mucosal layer up to the skin), size and conical elevation; this, in combination with salivary secretion, creates an assortment of different forms. The length of the canal, measured by fistulography, is 1 – 25 mm; the canal is often bifurcated and always ends as blind sacs surrounded by mucous glands [10]. These pits are mostly asymptomatic, or they can drain a small portion of saliva when provoked by mastication.

Diagnosis of VWS can be made clinically soon after birth for the majority of cases. Practitioners dealing with cleft patients must be aware of the syndrome's variety of expressions because early diagnosis is important for genetic counselling [4,8]. All VWS-affected parents should be cautioned that they carry a risk, even up to 50%, of having a child with a cleft lip and/or cleft palate [11].

The surgical treatment of rare cranio-facial syndromes could potentially encounter several obstacles. Firstly, the small number of treated cases and the rarity of the syndromes preclude reliable statistical evaluation of obtained results. The available information from literature comes from case reports rather than clinical indications based on reliable and statistically processed data. Secondly, these uncommonly performed procedures are unconventional because the syndromic cases are usually very complex and require the expertise of specialists from many fields of medicine. Furthermore, the lack of developed optimal treatment protocols increases the risk of potential complications. Finally, the success of treatment remains a contractual matter and can hardly be confirmed by any objective measure. Therefore, it is necessary to evaluate available material from as many cases as possible. The aim of this study is to evaluate our center's current surgical protocol for treating of patients with VWS, based on a series of consecutively treated patients in the years 2009–2022.

## Methods

This retrospective study, based on the medical database of a single institution, evaluated all patients born with cleft defects who were operated on in our center during 13 calendar years (2009–2022). The inclusion criteria were: 1) clinical diagnosis of VWS; 2) all surgical procedures performed at our center; 3) a follow-up of at least 6 months. The collected data were recorded and processed with Microsoft Excel 2019 for Windows. The analyzed variables included: patient`s gender, number and location of lip pits, type and extent of cleft defect, timing of lip pit excision as well as need for reoperation, and overall number of primary cleft repairs.

The primary cleft repairs were performed according to the one-stage surgical techniques described extensively in previous publications from our center [12,13,14].

In all cases lower lip pits repairs were performed under general anesthesia as a separate procedure in the surgical protocol. Due to variable forms, locations and sizes, each case necessitated a unique surgical technique in order to obtain the most favorable aesthetic outcome.

In most cases we performed a lenticular incision on the lip arriving at the bottom of the pit. Among patients with bilateral pits we had to choose between a double vertical incision or a single horizontal incision encompassing both pits. The main problem in large deformities was huge lip deficiency after the complete removal of pits. The shortening of lip length following the procedure was often accompanied by scarring causing bulbous lip widening. In such cases of deformation, subsequent corrective surgery was required and the final result was always uncertain.

## Results

The collected data comprised 24 patients diagnosed with VWS: 13 males (54%) and 11 (46%) females. The data of these patients are presented in Table 1. They suffered from the following types of cleft defects: bilateral cleft lip and palate (BCLP) – 6 patients; unilateral cleft lip and palate (UCLP) – 9 patients; cleft lip (CP) – 1 patient; and isolated cleft palate (CP) – 8 patients (Table 2).

**Table 1.** Material characteristics at the time of evaluation.

**No.**	**Patient initials**	**Gender (F/M)**	**Extent of cleft defect**	**Age at primary cleft repair (months)**	**Location of lower lip pits**	**Age at lip pits repair (months)**	**Necessity for reoperation**	**Overall number of primary repairs**
1.	MS	F	LASHSAL	8	bilateral	40		2
2.	TM	F	LASHSAL	10	unilateral	20		2
3.	DM	F	-hSh-	8	bilateral	85		2
4.	SM	M	-L-	7	bilateral	36		2
5.	WN	F	-hSh-	9	unilateral	48		2
6.	GS	M	LASH-	10	bilateral	23	yes	3
7.	WH	F	submucous	12	bileteral	24		2
8.	SA	M	LASH-	9	bilateral	22		2
9.	KD	F	-HSH-	8	unilateral	30	yes	3
10.	SD	F	LASHSAL	9	bilateral	24		3
11.	GN	F	LASHSAL	8	bilateral	14	yes	3
12.	ŻB	M	LASHSAL	9	unilateral	49		3
13.	TD	M	LASH-	10	bilateral	42	yes	3
14.	TZ	M	LASH-	9	bilateral	42	yes	3
15.	GK	M	-hSh-	7	unilateral	26	yes	3
16.	MK	F	LASH-	7	bilateral	60	yes	3
17.	MM	M	-hSh-	8	bilateral	18	yes	3
18.	WI	M	-hSh-	10	bilateral	24		2
19.	OJ	F	-SHAL	7	bilateral	26		2
20.	KJ	M	-hSh-	11	bilateral	23		2
21.	KM	M	LASHSAL	8	unilateral	60		2
22.	MW	F	-SHAL	9	unilateral	82	yes	3
23.	SM	M	LAHS-	7	unilateral	47		2
24.	NO	M	-SHAL	6	bilateral	24		2

**Table 2.** Breakdown of the different cleft types in the study group by gender and number of surgical.

**Cleft type**	**Male (%)**	**Female (%)**	**Number of surgical interventions**		

			**Mean**	**SD**	**Range**
cleft lip with or without cleft palate	9 (56)	7 (44)	2,5	0	2–3
isolated cleft palate	4 (50)	4 (50)	2,37	0	2–3
Total	13 (54)	11 (46)	2,46	0	2–3

In 16 (66%) cases, pits of the lower lip occurred on both side of the midline. In 8 (34%) cases, pits were detected unilaterally. The characteristics of the study group are presented in Table 1. The primary cleft repairs in the study group were performed according to the one-stage principle at the mean age of 8.6 months (SD 1.4, range 6–12) (Figs. 1, 2). In all cases, lower lip pits repairs were performed after the primary cleft repairs at the mean age of 37 months (SD 11.3, range 14–85). The mean number of all primary repairs of the syndrome, both cleft defect and lower lip pits repairs, was 2.5 (Table 2). In 9 patients (37.5%) additional secondary surgical corrections of the lower lip deformities were required due to the poor late post-operative cosmetic outcome (Table 1).

## Discussion

In this study we presented a series of patients suffering from VWS whose surgical treatments were performed at our center during a period of 13 years. The Institute of Mother and Child in Warsaw is the last reference cleft center in Poland and the largest cleft center in Europe, and performs approximately 480 primary cleft repairs per year. The extraordinary concentration of patients suffering from cranio-facial malformations at the Institute made it feasible to collect the study group of patients with as rare a syndrome as VWS during a relatively short period of time.

Children with lip or/and palate cleft are a very special group of patients and require and long-lasting care from multiple specialists, starting from the first days of their life. Surgical treatment plays a crucial role in obtaining satisfactory functional and aesthetic results for these patients.

The most common symptoms of VWS are bilateral sinuses of the lower lip, placed close to the midline symmetrically or asymmetrically. The pits can also be unilateral. The location, reach and depth of these lip pits vary from the mucosal layer up to the skin roll. This, the different sizes and conical elevations of the lip pits and salivary secretion all combine to create a variety of forms. The complexity of the patient`s disorders forces the surgeon to make decisions about their individual treatment scheme.

### Clinical relevance

Some authors recommend performing the lip pits excision and lip repair together with the primary cleft repair to reduce the overall number of procedures [15]. On the other hand, some complex cases with large fistulas, involving a significant part of the lip, could be divided into two or more procedures to achieve better cosmetic and functional effects. Although the procedure for lip pits in VWS appears straightforward, even in the best hands, the excision can be very challenging with no guarantee of aesthetically desirable results [15]. This study confirms the observations of other authors that poor aesthetic outcome of the primary surgical intervention into the lower lip in VWS often implies the need for two or more secondary surgeries due to residual deformities [3]. Secondary correction of post-operative deformities of the lower lip was necessary in 37.5% of the patients in the study group. Nevertheless, the average total number of primary surgical interventions in the study group was 2.46, a low number due to the one-stage surgical protocols for the treatment of cleft defects implemented in this cleft center. This should also explain why the long-lasting one-stage primary cleft repairs and the lower lip defects, as a rule, were performed as separate procedures. Cleft repair is performed via intraoral intubation with the endotracheal tube lying on the lower lip during the operation. Therefore, any surgical intervention into the lower lip would first require some additional maneuvers in order to relocate the endotracheal tube upwards from the region of the lower lip. These maneuvers combined with the time-consuming repairs of lower lip deformities would excessively extend the already long operating time. Furthermore, the edema of lower lip, often induced by the endotracheal tube position during the first part of the operation, could negatively influence the optimal outcome of subsequent lip pits repairs if performed one after the other.

The literature review shows that many authors assume that VWS has some gender predilection. Many authors believe that there is high prevalence of VWS in females nothing while others predominantly noted VWS in males [16]. The percentage of male and female patients in the present study is analogous to several other studies [8,17]. Gender predilection would more probably depend on a type of cleft defect (Table 2). Nevertheless, the small sample size does not allow for a deeper evaluation with a breakdown on cleft type related subgroups, which is one of the main limitations of the present study. At this point, we can only speculate that VWS could be more frequent in males suffering from cleft lips with or without cleft palates, while it might be more frequent in females with isolated cleft palates.

### Limitations

As a consequence of the young patients' age at evaluation (mean 4 years) and the diversity of included cleft defects, the study focused only on the primary repairs to the cleft and lip pits. Hence, secondary repairs (such as alveolar bone graftings, surgery for velopharyngeal insufficiency, fistula repairs, etc.) were not evaluated in this study. Another limitation of the study was its retrospective character, which is why the aesthetic results and subsequent re-operations were based only on medical records. Estimation of the patient's satisfaction was beyond the scope of this study. The limited number of participants in the study group precluded more profound statistical evaluation. However, these matters can be evaluated in future studies after collecting larger groups of patients with VWS.

## Conclusions

The present study registered the frequent need for secondary corrections of residual lower lip deformities following primary lip pits repairs among the patients suffering from VWS. This indicates the considerable difficulties in obtaining a satisfactory outcome of the procedure. As a rule, the primary cleft repairs and the lower lip pit repairs were performed as separate operations for all patients from the study group. Nevertheless, the average number of the primary surgical interventions registered in the study group including lower lip repairs was 2.46, and remained very low due to the one-stage surgical protocols for the treatment of cleft defects implemented in our center.
